# Impact of Applied Behavior Analysis on Autistic Children Target Behaviors: A Replication Using Repeated Measures

**DOI:** 10.7759/cureus.53372

**Published:** 2024-02-01

**Authors:** Tami Peterson, Jessica Dodson, Frederick Strale

**Affiliations:** 1 Hyperbaric Oxygen Therapy, The Oxford Center, Brighton, USA; 2 Applied Behavior Analysis, The Oxford Center, Brighton, USA; 3 Biostatistics, The Oxford Center, Brighton, USA

**Keywords:** target behaviors, discrete trial training, applied behavior analysis, repeated measures design, functional behavior assessment, autism spectrum disorder

## Abstract

Introduction: Applied behavior analysis (ABA) is a primary evidence-based practice in treating autism spectrum disorder (ASD). Ongoing research is needed to report the results of ABA relative to attaining target behaviors. This study aims to replicate the results of previous research to determine the effectiveness of ABA of target behaviors in autistic children with a new timepoint sample of data.

Materials & methods: A repeated measures analysis tracked 98 autistic children, which included four adult participants, over three timepoints during a one-month snapshot period from 6/7/23 to 7/7/23. This study used a retrospective chart review to gather data on target behaviors to determine the effectiveness of ABA treatments across age categories. A mixed (between x within) analysis of variance (ANOVA) and subsequent post hoc and interaction contrasts were used to determine statistical significance.

Results: Mixed (between x within) ANOVA indicated statistical significance (sphericity assumed), F(2,160) = 32.893, and p < 0.05, across time. Using bootstrapped paired t-tests, multiple comparisons indicated p < 0.001 on all three multiple comparisons, with Bonferroni corrected α = 0.017. There was also a non-significant interaction effect (sphericity assumed) with (time) x (age category), F(8,160) = 0.333, p = 0.952, likely due to sizeable within-group variation resulting in a lowered statistical power.

Conclusions: This replication found that autistic children receiving the ABA intervention demonstrated statistically significant improvement in target behaviors over the one-month snapshot period.

## Introduction

Background

Over 30 years of scientific research suggest that applied behavior analysis (ABA) interventions lead to evidence-based interventions supporting the development of individuals with autism spectrum disorder (ASD) [1-5]. Therapy using stimulus control (Sds), positive reinforcement (R+), the three-term contingency (antecedent, behavior, and consequence), and functional behavior assessment (FBA) are the basis of most effective ASD interventions [6]. Despite convincing evidence for the efficacy of ABA, concerns in the eyes of consumers exist which must be addressed [7]. The National Professional Development Center for Autism (NPDC), National Autism Center (NAC), Center for Medicare & Medicaid Services (CMS), and various current publications recommend that consumers of ABA educate themselves on the “practice” of ABA and what constitutes effective service delivery of evidence-based interventions [7,8]. 

The treatment of individuals with ASD is associated with fad, controversial, unsupported, disproven, and unvalidated treatments. Eclecticism is not the best approach for treating and educating children and adolescents who have ASD. ABA uses techniques derived from scientifically validated protocols. It incorporates treatments identified by the US National Research Council of the National Academies (NRC) as characteristic of effective interventional programs for children who have ASD. The only interventions shown to produce comprehensive, lasting results in autism have been based on the principles of ABA [8].

Peterson, Dodson, Hisey, Sherwin, and Strale [9] emphasized that "Discrete Trial Training is an applied behavior analytic modality that simplifies complexity by taking large, gross tasks, reducing them to small, individualized tasks, and teaching them with straightforward and systematic methods. Mass trials are a method within discrete trial training that includes repeatedly presenting the same stimulus until the learner responds correctly. Naturalistic environment training (NET) is a form of ABA that teaches behavioral skills within the natural context of a learning environment. The respective learner’s individual preferences and partialness serve as the motivation. The effects of a blend of discrete trial training, mass trials, and naturalistic environment training in autistic children are noteworthy as they can assist with various aspects of learner cognitive, language, social, and adaptive skills development. The benefits of discrete trial training include helping autistic children learn appropriate responses to different situations, which can enhance communication, their relationships with family, classmates, and peers, and overall quality of life. Acquisition of skills such as matching, discrimination, and imitation using this form of ABA can enhance learning that is difficult to acquire in naturalistic settings. Mass Trials assist autistic children with acquiring new behaviors more quickly and efficiently as exposure to the same or similar stimulus increases. This ABA method can help increase and retain learned behaviors over time by strengthening memory and improving recall abilities. NET assists autistic children with generalization skills transferred from discrete trial training to different contexts, including people, materials, and settings. NET also helps with increased motivation, spontaneity, and engagement by utilizing reinforcements that occur naturally and are aligned with learner interests [9]. The interventions using discrete trial training and mass trial interventions in a naturalistic environment, utilized in this study impacted academic, activities of daily living (ADLs), behavioral, expressive language, group, imitation, play, receptive language, self-regulation, self-management/cooperation, and social variables" [9].

Original study

Peterson, Dodson, Hisey, Sherwin, and Strale [9] reported on general ABA broad effectiveness with large N designs using a mixed behavioral model, e.g., discrete trial training, mass trials, and a naturalistic environment. Given the abundance of small N studies delineating the positive effects of ABA therapy, extensive large N studies of general ABA broad effectiveness can lead to further research to improve quality and outcomes. There is also a lack of published studies using repeated measures designs.

Peterson, Dodson, Hisey, Sherwin, and Strale [9] created the first piece of research utilizing a mixed model of ABA using discrete trial training, mass trials, and a naturalistic environment with a measured effect on general target mastery behavior information using a large N design with repeated measures. The statistical results showed that these interventions significantly increased general aggregate target behaviors over seven timepoints covering three months. We observed statistically significant increases in mean and median measurements of the number of multiple raters composite general target behaviors achieved per session. The multiple comparisons between timepoints indicated an upward trend of improvement and statistically significant differences between timepoints with medium to large effect sizes, the most prominent being in the age category of 13yrs.-16yrs.

Purpose of replication

The purpose of this study is to replicate Peterson, Dodson, Hisey, Sherwin, and Strale [9] in evaluating the effectiveness of ABA treatment in a retrospective snapshot cohort of n = 98 autistic children treated with ABA with functional analysis over three timepoints covering one month with a new dataset. It is hypothesized that the child cohorts treated with ABA will demonstrate statistically significant progress toward target behavioral goals, which will be documented and illustrated through tabular depictions of the magnitude of effects. 

The secondary objective of the study is to determine if an association exists between timepoints and age categories. It is hypothesized that the child cohorts treated with ABA will demonstrate statistically significant progress toward target behavioral goals and that the independent variable of time will significantly interact with age categories to produce overall significant effects between time within age categories.

Hypothesis

As repeated measures indicate, it is hypothesized that the child cohorts treated with the mixed model, consisting of discrete trial training and mass trial interventions in the naturalistic environment, will demonstrate statistically significant progress toward general target behavioral goals over the one-month time period.

Outline

The remainder of this replication study will consist of the materials and methods section which will discuss how the data was collected, a description of the ABA treatment administered (discrete trial training, mass trial interventions in the naturalistic environment), a description of the dependent and independent variables, the repeated measures design, retrospective power analysis, and mixed (between x within) analysis of variance (ANOVA) with step down analyses. The Results section will report findings relative to the descriptive statistics with the sample in terms of summary statistics (mean, median, SD, and range) as well as the descriptive statistics for the repeated measurements. The results of the mixed ANOVA will be reported along with the results of the step-down analyses (post hoc tests with bootstrapped paired t-tests with p-values and confidence intervals).

## Materials and methods

Participants and setting 

Target mastery data was collected for 98 autistic children, which included four adults greater than 18 years of age, via a retrospective chart review contained within the “Catalyst” tracking software, who were administered ABA treatment for one month between 6/7/23 and 7/7/23. All patients were seen and treated at The Oxford Centers (TOC; Brighton and Troy, Michigan, U.S.A). The Oxford Centers are outpatient facilities that provide various services for several conditions, including ASD. These services include ABA, nutrition therapy, neurofeedback, musical therapy, educational support, and HBOT. Children being treated TOC received any of these therapies. Data was collected via retrospective chart review, which was conducted to gather data relative to autistic children treated with ABA. Manuscript generation and reporting adhered to Consolidated Standards of Reporting Trials (CONSORT) and Strengthening the Reporting of Observational Studies in Epidemiology (STROBE) guidelines. 

Method of data collection and dependent variables with operational definitions 

The dependent variable was the number of target mastery behaviors achieved per session measured at three timepoints (Time 1-Baseline), (Time 2-At 2 Weeks), (Time 3-At 4 weeks). 

“Catalyst”, an ABA data collection software, generated automated progress reports for repeated measures outcome data for discrete trial teaching targets (DTT) with frequency and rate data. Mastery criteria for target behaviors were defined as the percentage of trials, minimum number of trials, and number of therapists above criteria. Graphs in Catalyst were customized to track progress and/or lack of progress with targeted behaviors. Catalyst automatically determined mastered targets as criteria are achieved. Three time variables (Time 1, Time 2, Time 3) were established as a snapshot of the child's progress or lack thereof for one month, beginning on 6/7/23 and running to 7/7/23. 

Experimental design-repeated measures over time 

This retrospective-repeated measures study assessed the clinical application of ABA with functional analysis [10] with the goal of increasing frequencies of target behaviors and decreasing problematic behaviors with a one-month snapshot from (6/7/23 through 7/7/23). Evaluation of the empirical effectiveness of treatments is more sensitive in repeated measure designs because they allow researchers to measure how the treatment affects each child. Repeated measures analysis deals with response outcomes measured on the same experimental unit at various times or under different conditions. In repeated measures designs, each subject serves as own control [11]. 

Repeated measures designs allow for the variability between subjects to be isolated, and analysis can focus more precisely on treatment effects. A repeated measures analysis puts each child on an equal footing and simply looks at how scores change with the same, similar, and/or alternative treatments over time [12]. 

Keselman et al. [13] noted that given the repeated-measure design is a research design in which subjects are measured two or more times on the dependent variable, rather than using different participants for each level of treatment, the participants are given the same and/or more than one treatment and are measured after each. This implies that each child was under its own control. Scores for the same child are considered dependent on each other. 

Because all children exposed to ABA (or who share a common characteristic) regardless of outcomes are included when reporting findings, they enable clinicians and researchers to identify functional relations that may have generality across separate cases and allow clinicians and researchers to determine their relative effectiveness. Identification of the variables that may possibly mediate the generality of ABA clinical procedures can be examined in subsequent analyses [14]. 

The reliability of the observed effects of ABA interventions in the context of an assessment procedure or treatment evaluation is important in the documentation of the experimental analysis performed. Reporting results from an analysis using repeated measures design structure and response patterns allows for detection of consistency and magnitude of effects, and documentation and the demonstration of experimental control. The summarization of ABA clinical outcomes data obtained from delivering standard ABA clinical care and the assessment or intervention selection is based on the clinician’s judgment about the needs of the individual child. 

Power analysis-study size 

A retrospective power analysis was conducted using G*Power 3.1 [15] and indicated that a total sample size of n = 27 participants would be required to demonstrate a high group effect size (.80) for a repeated measures design with nominal alpha (α) = .05 using a (between x within) repeated-measures ANOVA, with a power equal to 0.987. Given analysis parameters, there is a high likelihood that this current retrospective trial with n = 98 participants indicated an acceptable sample size criterion. 

Statistical methods 

IBM SPSS Statistics for Windows, Version 29 (Released 2023; IBM Corp., Armonk, New York, United States) was used for all descriptive and inferential analyses. Nominal alpha (α) was set at .05. If p-values are less than .05 (p < 0.05), a null hypothesis will be rejected, and statistical significance will be inferred. Demographics and baseline characteristics were summarized for all 98 subjects. Summary statistics for categorical variables, gender, and race/ethnicity, and for continuous variables, age, Time 1, Time 2, and Time 3 (mean and standard deviation, median, and range), will be generated. 

A mixed (between x within) ANOVA will be used to determine the overall statistical significance between the 3 (Time 1 to Time 3) levels of the independent dependent variable, as well as any Interaction effects between the fixed factor (Age) and the three (3) repeated measures timepoints assessing target behaviors. If an overall significant omnibus F statistic is detected (p < .05) within the Mixed (between x within) ANOVA, a step-down analysis will be performed using resampling multiple comparison procedures in the form of bootstrapped paired tests (1000 replications). Resampling methods using bootstrapping with the paired t-test mitigates potential multiplicity thereby reducing familywise error rate (FEW) likelihoods [16]. 

The Bonferroni correction will also be used as the as α = .05/3 = 0.017. Therefore, with these multiple comparisons, if p < 0.017, then a null hypothesis will be rejected, and statistical significance will be inferred [17]. 

If an overall significant omnibus interaction F statistic is detected (p < 0.05) within the mixed (between x within) ANOVA, a step-down analysis will be performed using Interaction Contrasts comparing the between subjects’ factor with the within subjects’ factor to determine specifically where the significant differences (effects) occurred.

Inter-observer reliability 

A two-way random effects model was computed where people's effects and measures effects are also random. We used ICC (2), which is used when multiple measurements are made from each averaged rater. The ICC (2) value was 0.929 (95% CI: 0.831-0.964), indicating excellent agreement between the raters. This value was larger than the average Pearson r (0.892), suggesting that the ICC (2) was equally sensitive to the variability among raters and measurements. Cronbach’s alpha for the three time point variables was r = .954 [9,10].

Institutional review board approval

Consent was obtained or waived by all participants in this study. The Oxford Center was issued approval number 1-170336-1 from WCG IRB. The authors declare that this research investigation involves minimal risk and complies with the Belmont Report Regulations 45 CFR 46 2018 Requirements (2018 Common Rule) Section 46 Subpart A Basic HHS Policy for Protection of Human Research Subjects, 46.104 Exempt Research Paragraph d (1), (2), and (2) ii and 46.117 Documentation of Informed Consent Paragraph c (1) (ii). This study also conformed to the guidelines outlined in the 1964 Declaration of Helsinki.

## Results

Demographics 

For the sample of 98 autistic children, the age was (M=9.0, SD =8.15), the median was 7.5, the minimum was 1, and the maximum was 73. There were 70 males (71.4%) and 25 females (25.5%), with three (3.1%) missing values. There were 68 whites (69.4%), 12 Asians (12.2%), five American Indian/Alaska Native (5.1%), four Hispanics (4.1%), and seven unspecified (7.1%), with two (2.0%) missing values. In terms of age categories, 17 (17.3%) were in the 1yr.-4yr. category, 37 (37.8%) were in the 5yr.-8yr. category, 20 (20.4%) were in the 9yr.-12yr. category, 12 (12.2%) were in the 13yr.-16yr. category, and 4 (4.1%) were in the 17yr.-73yr. category, with eight (8.2%) missing values. Please note that four subjects were over 17 years old, e.g., 18 yrs. old, 20 yrs. old, 25 yrs. old, and 73 yrs. old. Results for descriptive statistics relative to Time 1, Time 2, and Time 3 measurements are presented in Table 1.

**Table 1:** Descriptive statistics for the three timepoints (one-month snapshot)

Measure		Targets Mastered Baseline	Targets Mastered Two Weeks	Targets Mastered Four Weeks
N	Valid	93	93	93
	Missing	5	5	5
Mean		5.3226	9.7204	11.2903
Median		3.0000	6.0000	7.0000
Std. Deviation		8.97601	11.92218	12.63069
Minimum		0.00	0.00	1.00
Maximum		60.00	73.00	73.00

Assumptions for mixed (between x within) ANOVA 

The three timepoint measurements are scale variables, continuous (ratio/interval). The within-subjects factor consists of the same subjects measured at three timepoints (Time 1, Time 2, and Time 3) covering a one-month timeframe. 

The between-subjects factor should each consist of at least two categorical, "independent groups". Categorical independent groups in this study included age. 

There should be no significant outliers in any group of the between-subjects and within-subjects factor. Outliers are data points that do not follow the usual pattern. In this study, there exist outliers (above 99th %le in Boxplot), for Time 1 (Case #s 12, 31, 50, 68, 82 & 95), or 6.5% of the Time 1 data. For Time 2 (Case #s 36, 50, 51, 82, 83, & 95), or 6.5% of the Time 2 data. For Time 3 (Case #s 36, 41, 50, 51, 83, & 95), or 6.5% of the Time 3 data. Because of the nature of the learning progress of the population of autistic children, and this repeated measures analysis, the outliers will be retained as they are natural to the study’s research question. 

The dependent variable should be normally distributed for each combination of the groups of the two factors (i.e., within-subjects factor and between-subjects factors). To test for normality, we examined the Q-Q plots and found non-alignment. Also, the skewness scores for all three timepoint datapoints were all outside the typically accepted range, of -1 to +1 (M = +3.24, SD = 0.250). Mixed (between x within) ANOVA is quite "robust" to violations of normality, meaning that the assumption can be violated and still provide valid results. 

Homogeneity of variances for each combination of the within-subjects factor and between-subjects factors is required. “Sphericity” relates to the variances of the differences between the related groups of the within-subject factor for all groups of the between-subjects factor (the within-subjects factor and between-subjects factor) must be equal. 

Mauchly’s test of sphericity indicates the assumption of sphericity has not been met, Mauchly’s W = 0.226, Approximate Chi-Square = 117.431, df = 2, p < 0.001, Greenhouse-Geyser Epsilon = 0.564, Huynh-Feldt Epsilon = 0.595, Lower-bound = 0.500. Greenhouse-Geyser Epsilon will be used to adjust the degrees of freedom for the averaged tests of significance [18]. Corrected tests (F-values) are reported in the Results section. Several investigations [18,19] using Monte Carlo Simulations into the robustness of generalized linear models (GLMs) of which mixed (between x within) ANOVA is a member, have been reported, suggesting robustness (the likelihoods of Type I error are reduced). 

Main effects of mixed ANOVA (between x within) - 

There was a significant main effect (sphericity assumed) on the dependent variable (targets mastered) across Time, F(2,160) = 32.893, p < 0.001). This indicates an overall statistically significant effect (increase in targets mastered) detected across the three timepoints of the independent variable (time) over the one-month period. 

Post hoc analyses 

Post hoc analysis was conducted using Bootstrapped (1000 replications) paired t-tests for multiple comparisons with a Bonferroni corrected (.05/3) α = 0.017. Results indicated that for all three timepoint comparisons (Time 1 vs Time 2), (Time 1 vs Time 3), and (Time 2 vs Time 3), across the time dimension, all p-values were < 0.05 and were statistically significant at α = 0.05 (see Table 2).

**Table 2:** Bootstrapped (1000 replications) paired t-test for multiple comparisons

Outcome Variables		Mean	Bootstrap				
			Bias	Std. Error	p-value	95% Confidence Interval	
						Lower	Upper
Pair 1	Targets Mastered Baseline - Targets Mastered 2 Weeks	-4.39785	-0.01960	0.62959	< 0.001	-5.68817	-3.25861
Pair 2	Targets Mastered Baseline - Targets Mastered 4 Weeks	-5.96774	-0.02989	0.76628	< 0.001	-7.52661	-4.58065
Pair 3	Targets Mastered 2 Weeks - Targets Mastered 4 Weeks	-1.56989	-0.01029	0.28374	< 0.001	-2.17204	-1.07554

Mixed ANOVA - interaction effects - time x age category

There was also a non-significant interaction effect (Sphericity assumed) with (Time) x (Age category), F(8,160) = 0.333, p = 0.952), likely due to a sizeable within-group variation resulting in a lower statistical power. 

## Discussion

Summary of findings

The statistical results suggested that ABA intervention over three timepoint measurements significantly increases aggregate target behaviors. Expressly, the multiple comparisons between each time point indicated an upward trend of improvement and statistically significant differences between time points. This hypothesis was confirmed. 

The study's secondary objective was to determine if an association existed between the three time points and age categories. It was hypothesized that the child cohorts treated with ABA would demonstrate statistically significant progress toward target behavioral goals. The independent variable of time would significantly interact with age categories to produce overall significant effects between time within age categories. This hypothesis was not confirmed in this investigation. 

Comparison with the original study

This study found similar results to the statistically significant findings of Peterson, Dodson, Hisey, Sherwin, and Strale [9]. We evaluated the effectiveness of ABA treatment in a retrospective snapshot cohort of n = 98 autistic children treated with ABA with functional analysis consisting of discrete trial training, and mass trials, in a naturalistic environment over three time points covering a one-month snapshot. Unlike the original study, however, there was a non-significant Time x Age interaction. 

Implications

This study increases confidence in the results of our original study. As noted by Peterson, Dodson, Hisey, Sherwin, and Strale [9], discrete trial training reduces large tasks to smaller, more manageable tasks and teaches these tasks with straightforward and systematic methods. Within discrete trial training, mass trials emphasize repeated stimuli presented until the learner performs the task correctly. NET trains behavioral skills within a natural environment, with individual preferences as the motivation to learn. The multimodality of these three treatments with autistic children is noteworthy in assisting with learners' cognitive, language, social, and adaptive skills development. Discrete trial training is beneficial in helping autistic children learn different responses to varying situations, enhancing communication, family, peer, and classmate relations, and overall quality of life. Skill acquisition such as matching, discrimination, and imitation using this form of ABA can serve to enhance learning that is difficult in naturalistic settings.

Mass trials help autistic children acquire new behaviors efficiently and quickly with the same or similar stimulus exposure increasing. Increase and retention of learned behaviors increase over time and strengthen memory and recall abilities. NET helps autistic children with generalization skills transferred from discrete trial training to different contexts, including people, materials, and settings. NET also helps with increased motivation, spontaneity, and engagement by utilizing reinforcements that occur naturally and are aligned with learner interests [9].

The steady increase in this replication study with general target mastery behaviors over the designated three time points covering one month is noteworthy. Ongoing studies of general ABA broad effectiveness, namely, with discrete trial training, mass trials, and naturalistic environment training, with Large N studies, can lead to further research to improve quality and service.

This replication study continued to address consumer concerns and misconceptions and inform consumers on the “practice” of ABA and the effective service delivery of evidence-based therapies as requested by the NPDC [20]. This research is consistent with NPDC, the NAC, and various current articles advising consumers of ABA to become well-versed on the “practice” of ABA and the characteristics of effective service delivery of evidence-based behavior analytic interventions. These recommendations can continuously inform families, educators, clinicians, and policymakers about the benefits and limitations of ABA with autistic children. Research must continue to support evidence-based practices and continued improvement [21].

Limitations

Although the results of this replication are informative, it has limitations. Again, a convenience sample was used. There is no ability to generalize beyond this sample. Due to data constraints, there is no delineation of possible statistically significant differences between the groups relative to discrete trial training, mass trials, and NET.

Seven threats to internal validity apply in pre-experimental research designs of this type. History points toward extraneous variables not part of the study or any external events that may affect outcomes. Maturation involves age-related bodily changes and includes age-related physical changes that can occur with time, such as hunger, tiredness, fatigue, wound healing, surgery recovery, disease progression, etc. Testing relates to the notion that the test may affect the children’s responses when tested again. These are less of an issue when the tests are routine. Instrumentation refers to any change in measurement ability, including any judge, rater, etc. Statistical Regression is the tendency for individuals who score extremely high or low on a measure to score closer to the mean of that variable the next time they are measured on it. Selection refers to the potential bias in selecting participants who will serve in the experimental and control groups. Mortality refers to the differential loss of study participants, drop-out rate, or attrition [22].

This is a within-subjects design, whereby each subject serves as its own control. Ethical issues preclude utilizing a control group (no treatment) for autistic children. In addition, this is a snapshot study covering one months. It will be informative to assess these children over a longer time longitudinally. This paper uses retrospective data, and while the single group pre-post design is pre-experimental, a prospective study is warranted for future investigation.

There appears to be a need in the literature concerning the analysis of discrete trial training and naturalistic environment training with repeated measures that call for future inquiry [5,9].

## Conclusions

Our replication found that autistic children receiving the ABA intervention demonstrated statistically significant improvement in target behaviors over the one-month snapshot period. Also, a non-significant interaction was found between the independent variable (time) and the age category on target behaviors, suggesting no association between time and age in this study. This is a replication of the first piece of research utilizing a mixed model of discrete trial training, mass trials, and a naturalistic environment with a measured effect on general target mastery information using a large N design with repeated measures. The statistical results showed that these interventions significantly increased general aggregate target behaviors over three timepoints. We observed statistically significant increases in mean and median measurements of the number of multiple raters composite general target behaviors achieved per session. The multiple comparisons between time points indicated an upward trend of improvement and statistically significant differences between timepoints.
